# Recent updates on the influence of iron and magnesium on vascular, renal, and adipose inflammation and possible consequences for hypertension

**DOI:** 10.1097/HJH.0000000000003829

**Published:** 2024-09-11

**Authors:** Benjamin J. Connolly, Sophie N. Saxton

**Affiliations:** Divison of Cardiovascular Sciences, The University of Manchester, Manchester, UK

**Keywords:** adipose, hypertension, inflammation, iron, kidney, magnesium, micronutrient, obesity, renal, vascular

## Abstract

The inflammatory status of the kidneys, vasculature, and perivascular adipose tissue (PVAT) has a significant influence on blood pressure and hypertension. Numerous micronutrients play an influential role in hypertension-driving inflammatory processes, and recent reports have provided bases for potential targeted modulation of these micronutrients to reduce hypertension. Iron overload in adipose tissue macrophages and adipocytes engenders an inflammatory environment and may contribute to impaired anticontractile signalling, and thus a treatment such as chelation therapy may hold a key to reducing blood pressure. Similarly, magnesium intake has proven to greatly influence inflammatory signalling and concurrent hypertension in both healthy animals and in a model for chronic kidney disease, demonstrating its potential clinical utility. These findings highlight the importance of further research to determine the efficacy of micronutrient-targeted treatments for the amelioration of hypertension and their potential translation into clinical application.

## INTRODUCTION

Hypertension, defined by the World Health Organization as a consistently elevated blood pressure (BP) of 140/90 mmHg or higher, is a major risk factor for numerous cardiovascular complications including angina, myocardial infarction, heart failure, arrhythmias, and stroke, as well as renal damage and dysfunction [1] Globally, around 1.28 billion adults (over 1 in 8) between 30 and 79 years of age exhibit hypertension, approximately 80% of cases of which are uncontrolled [1].

The condition, which is a contributing factor in approximately 13.5% of annual global deaths [2] can originate from one or a combination of pathologies. These various pathologies, which highlight the condition's complex nature, include aberrant renin-angiotensin–aldosterone signalling, overproduction of vasoconstrictors or underproduction of vasodilators, lesions in vasculature and microvasculature, which can be generated by oxidative stress, and inflammation [3,4]. The influence of inflammatory signalling on hypertension has been recognised as early as 1986, when Bataillard *et al.*[5] observed that thymectomy in hypertensive rats elicited an antihypertensive effect. Subsequent years of research have revealed that almost all potential origins of hypertension are intrinsically linked to inflammation [6]. This evidence accentuates the close association between inflammation and hypertension and the potential to treat hypertension through targeting inflammation.

Since research into inflammatory signalling is already well established, there is now potential to treat hypertension through targeting known inflammatory pathways that contribute to the condition. Recently, some exciting studies [7,9] have emerged detailing the impact that micronutrients can have on inflammatory factors, such as macrophage polarisation or inflammatory cytokine expression. These studies report that altered micronutrient metabolism can either ameliorate or exacerbate inflammatory signalling, depending on the micronutrient and its cellular interactions. This review will cover studies from the past two years, whose findings concerning the influence of iron and magnesium (Mg) on inflammation and the possible consequences for hypertension suggest a prominent role for these micronutrients in BP control [7,9]. Numerous other micronutrients such as vitamin D, zinc and sodium have also been shown to influence inflammation in hypertension and have been well reviewed [10–13]. However, because no studies have provided any updates on the influence of these micronutrients in inflammation in hypertension within the last 3 years, they have not been explored in this review.

This review explores recent reports regarding the interplay between cellular manipulation of iron and Mg, inflammatory signalling, oxidative damage and hypertension. It also examines how these updates may provide a foundation for developing micronutrient-targeted treatments for the alleviation of hypertension.

## AN OVERVIEW OF THE LINK BETWEEN INFLAMMATION AND HYPERTENSION

The causal link between inflammation and hypertension has been documented and built upon in medical research for well over 30 years [5]. The multifaceted response to damage and disease factors involves an array of immune cells, inflammatory cytokines and receptors, adhesion molecules and structural changes [6,14]. In health, acute inflammationserves as a healthy response to tissue damage or the invasion of a pathogen that allows for damage to be repaired, foreign bodies to be eliminated and tissue homeostasis to be restored. However, various factors can lead to the onset of *chronic* inflammation, which can become systemic and lead to multiple organ damage and dysfunction and fibrosis. In the context of hypertension, virtually all elements of inflammation have been reported to be involved in the onset of the condition, and thus the development of treatments targeting inflammatory pathways is paramount in exploring new avenues for the remediation of hypertension [6,15]. The numerous mechanisms by which inflammation causes hypertension have been extensively reviewed [6,16,17], and these will be briefly discussed here before covering in depth the contribution of iron and Mg to this process.

**FIGURE 1:**
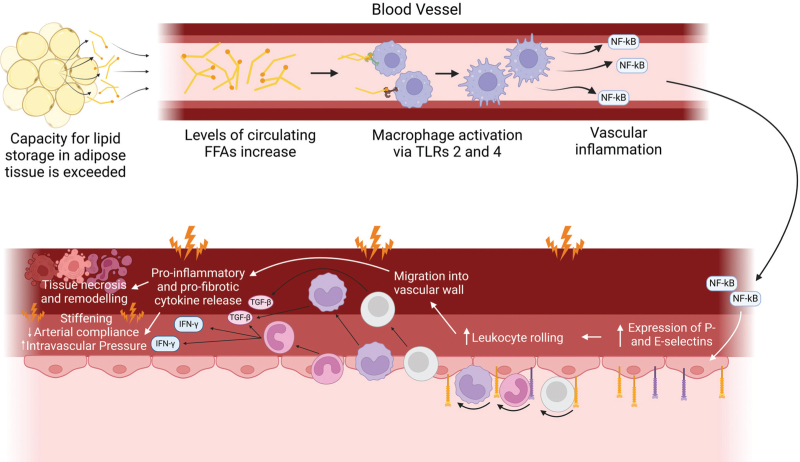
Over-saturation of lipid stores in obesity contributes to vascular inflammation, which leads to hypertension. Adipose tissue can only store a finite volume of lipids – when this volume is exceeded, lipids begin to accumulate in high concentrations in the circulation as free fatty acids (FFAs). These circulating FFAs interact with toll-like receptors (TLRs) -2 and -4 on proximal macrophages, leading to their activation. When activated, these macrophages cause surrounding vascular cells to express nuclear factor κβ (NF-κβ), inducing an inflammatory state in the vasculature. Once an inflammatory state has arisen in the blood vessel wall, the cell adhesion molecules E- and P-selectin are up-regulated, causing leukocytes to bind and roll along the inner surface of the vessel wall before extravasating into the surrounding tissue. Here, they release pro-inflammatory and pro-fibrotic cytokines, causing necrosis and tissue remodelling respectively. This causes the blood vessel wall to stiffen, resulting in reduced arterial compliance and increased blood pressure. Figure created with BioRender.com.

Chronic inflammation often accompanies obesity, and this can significantly contribute to the onset of hypertension in obese patients. In the obese state, the capacity limit for lipid storage in adipocytes is exceeded, leading to elevated levels of circulating free fatty acids (FFAs) [18,19]. These plasma FFAs promote inflammatory signalling through interactions with macrophages via toll-like-receptors (TLRs) -2 and -4 [20–22], which stimulatethe expression of nuclear factor κβ (NF-κβ) in the surrounding vasculature, generating inflammatory conditions [23]. This process is illustrated in Fig. 1.

Inflammation in the vasculature stimulates the expression of various adhesion molecules and chemokines, allowing for adhesion, rolling and migration of leukocytes into the vasculature. Here, leukocytes release the pro-inflammatory cytokine interferon γ (IFN-γ), which further promotes inflammation and induces necrosis, and transforming growth factor β (TGFβ), a fibrotic cytokine that induces tissue remodelling [6]. The resulting organisational disruption induces stiffening of vessel walls, leading to reduced arterial compliance and increased intravascular pressure, generating pathologically elevated BP [16,24]. A summary of this process is shown in Fig. 1.

FFAs also contribute to inflammatory signalling through inducing NADPH oxidase activity and the downregulation of antioxidant gene expression, increasing reactive oxygen species (ROS) production and activity respectively [17,25]. ROS activity leads to T-cell activation and severely alters inflammatory homeostasis. In endothelial, vascular smooth muscle and renal cells, ROS promotes the production of pro-inflammatory factors, particularly NF-κβ and matrix metalloproteinases [6,17,26,27]. In adipose tissue, production of anti-inflammatory adipokines such as adiponectin are significantly decreased in the presence of ROS, whilst the pro-inflammatory cytokine interleukin (IL)-6, is upregulated [25–30]. This shift towards pro-inflammatory signalling can then promote further ROS production, generating a cycle of ROS production, inflammation and vascular damage [19,31,32].

Inflammation in adipose tissue can also precipitate hypertension. In obesity, adipose tissue infiltration by pro-inflammatory M1 macrophages is extensive, in part due to hypoxia-induced inflammatory signalling resulting from adipose tissue expansion. Despite excessive hypertrophy of adipose tissue, there is minimal accompanying angiogenesis, resulting in hypoxic regions where inflammation arises [30,32,33]. In response, M1 macrophages infiltrate the tissue and release pro-inflammatory cytokines such as tumour necrosis factor (TNF)-α and ILs-1β and 6, which exacerbate the inflammatory state [29,32]. The recruitment of macrophages and their induction to the inflammatory M1 phenotype involves a plethora of immune cells. Dendritic cells are involved in disrupting the equilibrium between T-regulatory and Th17 cells, leading to a pro-inflammatory shift in T-helper cell phenotype and driving M1 polarisation in adipose tissue macrophages (ATMs) [34,35], while mast cells are implicated in ATM infiltration in obese mice [36]. CD4^+^ and CD8^+^ T-cells, whose major secretory product IFN-γ promotes M1 ATM polarisation [37–39], and B-cells, whose IgG2c antibody secretions induce inflammation in adipose tissue of lean mice [40] and have been suggested to play a role in M1 ATM polarisation [35,41], are also implicated in the M1 polarisation pathway. Additionally, neutrophils are rapidly recruited to adipose tissue following weight gain, and these granulocytes are also involved in maintaining inflammatory signallingpolarisation [20,42,35].

Perivascular adipose tissue (PVAT), which surrounds most blood vessels in the body [43], often becomes inflamed in obesity, resulting in high levels of ATM infiltration and white blood cell activity. In nonobese individuals, PVAT elicits an anticontractile effect on the vasculature primarily through the release of adiponectin, which induces the production and release of nitric oxide (NO) in an eosinophil- and endothelium-dependent manner [30,44–47]. In obesity, however, adiponectin release is impaired by hypoxia-induced inflammation, driven by the presence of M1 macrophages and supporting immune cells. This impaired release of adiponectin abolishes the anticontractile effect of PVAT and gives rise to increased blood vessel constriction and elevated BP [48] (extensively reviewed by Fernández-Alfonso *et al.*[49] and Saxton *et al.*[50]). This phenomenon signifies a strong correlation between inflammation in PVAT and hypertension, and that inflammation in adipose tissue is a promising therapeutic target for controlling hypertension in obesity. Recent evidence suggests that adipose tissue inflammation is substantially influenced by intracellular and mitochondrial homeostasis of iron, which may mean that iron manipulation in PVAT influences the tissue's ability to exhibit an anticontractile effect, and thus linked to hypertension [7]. This potential association is explored in more detail below.

Chronic inflammation is also associated with the presence and progression of chronic kidney disease (CKD) [51]. The condition, which is characterized by consistently impaired blood filtration by the kidneys, gives rise to the circulation of a multitude of pro-inflammatory molecules in the blood [52]. The body's inability to excrete these deleterious factors contributes extensively to inflammatory signalling in the vasculature [53], which can then contribute to hypertension. Reduced kidney function also results in increased blood volume, which elevates BP [54]. In summary, CKD is a major contributor to hypertension, both through chronic vascular inflammation resulting from persistent elevation of inflammatory molecules in the serum and through increased blood volume [16,24,54].

Broadly, inflammation is a complex and hugely influential contributor to hypertension. Its many facets give rise to myriad opportunities for exogenous modulation of the inflammatory process and therefore provide multiple options for treating inflammatory diseases, including hypertension. In theory, nonsteroidal anti-inflammatory drugs (NSAIDs) should serve as an ideal treatment for inflammatory hypertension due to their ability to inhibit inflammatory processes – indeed, the cyclo-oxygenase-2 inhibitor celecoxib has been shown to improve endothelial function in multiple studies [55–57]. However, they also have a pronounced effect on renal function. NSAIDs reduce glomerular filtration rate and inhibit sodium and potassium excretion, leading to fluid retention, increased blood volume and heightened BP [58,59]. Any beneficial effect on hypertension that arises from the anti-inflammatory properties of NSAIDs is therefore negated by their effect on renal function. Additionally, NSAIDs can exacerbate hypertension in hypertensive patients [60], rendering them ineffective as antihypertensive medication. Evidently, a new approach to targeting the inflammatory origin of hypertension is necessary.

Recent reports have detailed the manners in which micronutrients such as iron and Mg influence inflammatory signalling and may therefore contribute to the exacerbation or amelioration of hypertension, without the negative effects seen in NSAIDs [7–9]. This review has explored these contributions, their influence on the inflammatory process, and their potential for utilisation for the treatment of hypertension.

## IRON OVERLOAD IN ADIPOSE TISSUE MACROPHAGES IN OBESITY INDUCES INFLAMMATORY SIGNALLING, WHILST IRON DEPLETION ALLEVIATES INFLAMMATION

In the healthy, nonobese state, white adipose tissue comprises a subset of ATMs that are specially adapted for maximally efficient iron processing (MFe^hi^ cells) through increased expression of genes involve in iron uptake, metabolism, storage and release [61]. These ATMs display a phenotype akin to that of anti-inflammatory M2 macrophages [62]. However, in the obese state, hepcidin, the predominant regulator of iron metabolism in tissues, is pathologically upregulated, inhibiting cellular iron efflux in these iron-processing ATMs [63]. Hepcidin upregulation induces MFe^hi^ cells to transition to a pro-inflammatory M1 phenotype and sequester iron, whilst also bringing about the recruitment of ATMs with a reduced capacity for iron processing (MFe^lo^) [64]. This increase in MFe^lo^ cells means that the proportion of MFe^hi^ cells with regard to total ATM content is reduced [7].

**FIGURE 2:**
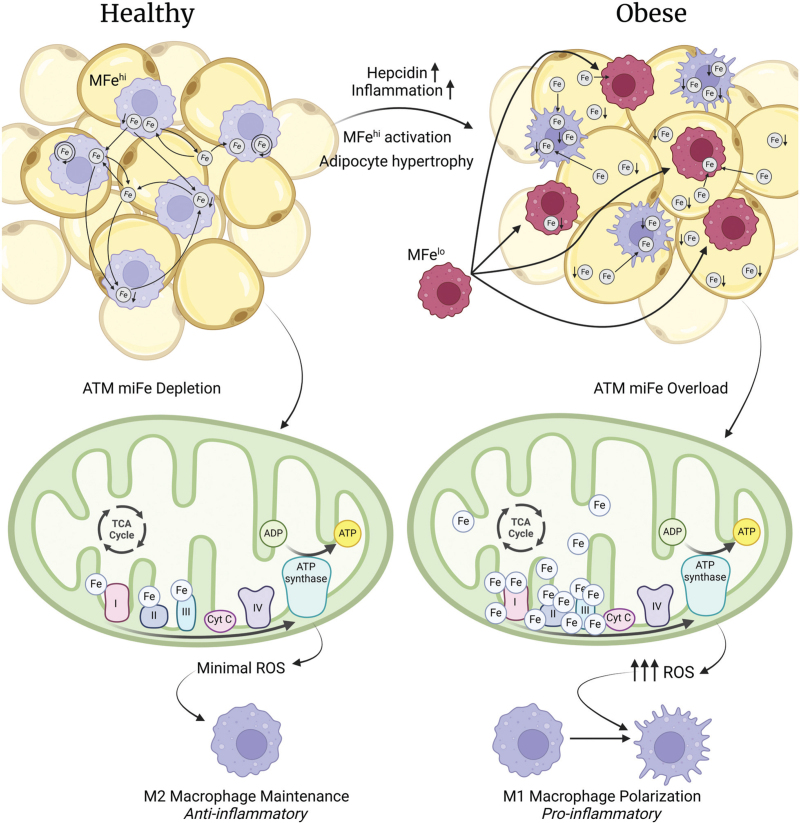
The inflammation-driving changes in cellular and mitochondrial iron regulation in adipose tissue macrophages (ATMs) in obese conditions. In healthy tissue, macrophages specially adapted for efficient iron processing (MFe^hi^) display high expression of genes involved in iron import, storage metabolism and export. This allows for heathy homeostasis of iron. In obesity, hepcidin expression and inflammation increase, causing alterations in the expression of genes involved in iron processing and the recruitment of macrophages which cannot process iron efficiently (MFe^lo^). As a result, iron uptake is increased, while its storage, metabolism and export are reduced. Iron homeostasis becomes significantly impaired, causing adipocytes to store excess labile iron. This iron can accumulate in the mitochondria where it binds to complexes I, II and III of the electron transport chain, increasing its activity and stimulating excess reactive oxygen species (ROS) production. The increased presence of ROS then stimulates the polarisation of M1 macrophages, which possess a pro-inflammatory phenotype. Figure created with BioRender.com.

**FIGURE 3:**
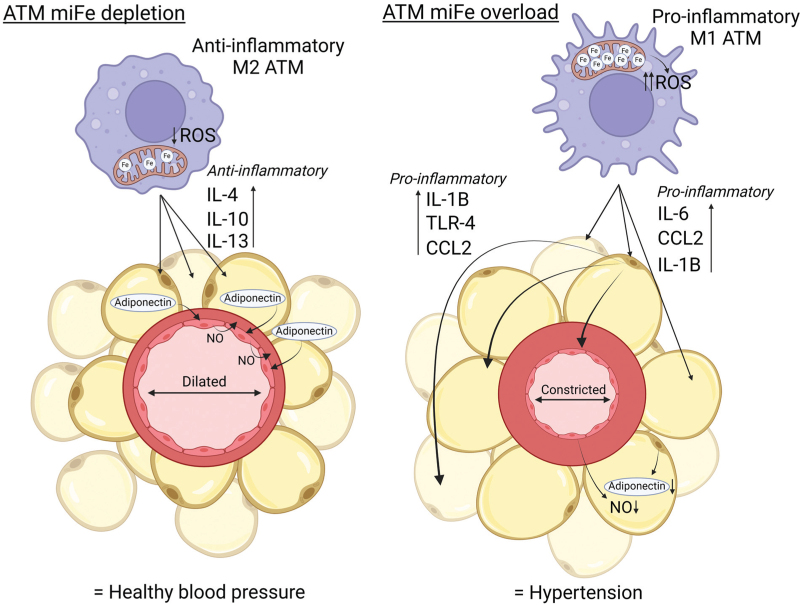
The proposed mechanism in which mitochondrial iron (miFe) depletion may contribute to reduced blood pressure (BP). Where miFe is depleted in adipose tissue macrophages (ATMs), reactive oxygen species (ROS) production is low, and the ATMs exhibit an anti-inflammatory profile. The anti-inflammatory interleukins (ILs) -4, -10 and -13 are released, promoting an anti-inflammatory state in surrounding adipocytes. This allows them to express beneficial levels of adiponectin, which ultimately stimulates nitric oxide (NO) release by vascular endothelial cells, leading to vasorelaxation and reduced blood pressure. However, where there is an overload of miFe in ATMs, pro-inflammatory molecules are released by both ATMs and adipocytes due to increased ROS, namely interleukins (ILs) -6 and -1β, toll-like receptor (TLR)-4 and chemokine (C-C motif) ligand (CCL)2. There is also a reduction in the production of the anti-inflammatory ILs -4 and -10, as well as adiponectin, by ATMs and macrophages. This reduction in adiponectin means NO production by endothelial cells is greatly reduced, resulting in vasoconstriction and increased blood pressure. Figure created with BioRender.com.

As a result, iron begins to accumulate and becomes sequestered in the cytosol and mitochondria of ATMs. The overload of iron in ATMs causes them to lose their buffering capability, consequently resulting in iron overload in neighbouring adipocytes and their mitochondria and generating inflammation and dysfunction in adipose tissue. [65,66]. This relationship, which is illustrated in Fig. 2, between iron overload and adipose tissue inflammation, suggests that there could be a link between iron overload and the anticontractile effect of PVAT, since ATMs are just as present in PVAT as in all other types of adipose tissue [49,50]. This potential link suggests that iron overload may abolish the anticontractile effect of PVAT and contribute to hypertension – this possibility is illustrated in Fig. 3. Indeed, iron overload has been associated with insulin resistance, metabolic syndrome, and obesity in humans, all of which are linked to hypertension [67,68]. Additionally, the reduction of whole-body iron stores via phlebotomy in humans has been clinically proven to reduce blood pressure and blood glucose [69], although current evidence does not yet indicate whether this reduction impacts iron stores in ATMs and adipocytes. Currently, there is no direct correlation between iron overload in ATMs and hypertension, but evidence linking ATM iron overload with inflammation, impaired adiponectin release, metabolic syndrome and obesity suggests that there could be and as-of-yet undiscovered link [60,70,71]. The potential for iron metabolism to modulate blood pressure is an exciting prospect for hypertension treatment. Joffin *et al.*[7] recently explored the effect of dysfunctional iron metabolism in adipose tissue on inflammation and the resulting changes in inflammatory signalling, glucose metabolism and body weight, providing compelling evidence for a potential link between iron overload and hypertension.

The study covered in this review [7] does not refer specific oxidation states of iron, but refers to analyses of both ferritin and transferrin. The former binds Fe^2+^ and stores it as Fe^3+^ through ferroxidase activity, whilst the latter solely binds Fe^3+^[72,73]. Any reference to iron in this review therefore primarily pertains to iron in its +3 oxidation state.

Adipocytes of mice fed a high-fat diet (HFD) display upregulation of the iron uptake receptor CD91, a marker of a pro-inflammatory phenotype, whilst CD163, a receptor of the same family that indicates an anti-inflammatory state, and the iron exporter ferroprotein, are downregulated [7,74]. The resulting uptake and reduced export of iron leads to a pathological accumulation of iron in the adipocytes of HFD-fed mice. ATMs from the same mice displayed reduced levels of MitoNEET, a mitochondrial protein involved in restricting the entry of iron into the organelle, and increased levels of mitochondrial ferritin (FTMT), involved in mitochondrial iron (miFe) import [75]. Decreased expression of MitoNEET also occurs in adipocytes of HFD-fed mice, confirming that iron overload occurs in the mitochondria of both ATMs and adipocytes in the obese mouse phenotype [7]. Obesity-induced loss of mitoNEET is also present in obese humans [76] suggesting that excess iron deposition in human adipocyte and ATM mitochondria may occur in obesity, with subsequent effects on inflammation and potentially hypertension. Using a transgenic HFD-fed mouse model, Joffin *et al.*[7] were able to selectively upregulate FTMT or mitoNEET, leading to either miFe overload or depletion respectively. The group demonstrated that ATMs with iron-overloaded mitochondria display increased expression of multiple pro-inflammatory factors, including chemokine (C–C motif) ligand 2 (CCL2), ILs -1β and -6, and reduced expression of the anti-inflammatory ILs -4 and -10, suggesting iron levels in mitochondria play a vital role in local inflammatory signalling in the obese state. Similarly, in adipocytes, increases in IL-1β, CCL2 and TLR-4 were observed. Importantly, a decrease in adiponectin expression and secretion in adipocytes was reported, reinforcing the potential for an association between miFe overload and hypertension through a reduction in vasodilator adipokines [7,77]. These findings affirm those reported by Zheng *et al.*[78], Hubler *et al.*[79] and Gao *et al.*[80], which also support the link between increased ATM miFe levels and the promotion of local inflammation.

On the other hand, ATMs from HFD-fed mice with depleted miFe (through upregulation of mitoNEET) exhibit an anti-inflammatory gene expression profile, despite the presence of obesity. This profile includes decreased expression of the inflammatory markers Cd11c, IL-6, TNF-α and IL-1β, and increased expression of anti-inflammatory ILs -4, -10 and -13 [7]. Reductions in the total ATM population and the ratio of M1 to M2 macrophages were also reported. MiFe depletion was also shown to decrease ROS levels through reduced lipid peroxidation and protein carbonylation [7], highlighting not only an anti-inflammatory effect of miFe depletion, but an antioxidant effect too. The group reported that iron-depleted ATMs exerted protective effects on surrounding adipocytes via paracrine anti-inflammatory signalling, allowing for healthy adiponectin production [7]. This highlights the importance of secreted products from ATMs in protecting adipocytes against iron overload. Although BP was not measured in mice with upregulated mitoNEET, it could be likely that iron depletion in adipose tissue has an effect on BP due to the influence of PVAT on hypertension. If miFe were to be depleted in hypertrophic and inflamed PVAT, alleviating inflammation in the tissue, the anticontractile effect of PVAT may be partially restored, and a subsequent decrease in BP may follow [81], as shown in Fig. 3. However, further research into the effect of miFe depletion on BP is necessary to determine the involvement of miFe in BP modulation.

Inflammatory changes resulting from miFe depletion in the obese mouse model are not isolated at the cellular level; Joffin *et al.*[7] reported favourable effects at the whole-organism level and for a prolonged period. After 6 weeks of miFe depletion, obese mice exhibited drastically reduced body weight and total body fat, suggested to be due to a preferential switch from carbohydrates to lipids as a primary energy source. Histological markers of inflammation in adipose tissue such as crown-like structures were also reduced, and adiponectin and leptin levels in serum were raised. Fasting serum insulin and adipocyte size were reduced, whilst glucose tolerance and insulin sensitivity were improved, and the pro-inflammatory markers TNF-α, IL-6 and IL-1β decreased. The anti-inflammatory markers IL-13 and the proportion of M2 macrophages were increased [7]. This evidence highlights not just the protective effects of iron depletion, but the ability to rescue inflamed adipose tissue. This may potentially restore the anticontractile effect of PVAT and hold potential for treating obesity-related hypertension.

The inflammatory benefits resulting from an iron-depletion-induced reduction in mitochondrial metabolism can be attributed to the iron content in the mitochondrial proteins involved in the electron transport chain (ETC) [7]. Iron is enriched in complexes I, II and III of the ETC, and this renders it a significant modulator of mitochondrial function and metabolism. When mitochondria are overloaded with iron, these complexes can begin to produce excess ROS [70]. This process is illustrated in Fig. 2. Studies have shown that inhibiting ROS production in complex I promotes the switching of macrophages from the M1 to the M2 phenotype [82,83] and thus promotes an anti-inflammatory environment, and the findings reported by Joffin *et al.*[7] ratify this finding. Increased activity in complex II has also been shown to be partially responsible for M1 macrophage activation and IL-1β production [84,85]. This evidence provides a mechanism of action for miFe depletion and the subsequent healthy functioning of ATM mitochondria, providing a solid basis for further research into a link between miFe depletion and inflammatory hypertension.

A possible theory for the basis of the detrimental effect of miFe overload can be drawn from evidence showing that chelation of iron in macrophages reduces oxidative metabolism and thus prevents the production of ROS and subsequent pro-inflammatory signalling [84,86]. An excess of miFe may mean that there is too much iron present to be naturally chelated in cells, meaning the data presented by Joffin *et al.*[7] may be explained by a decrease in the ratio of chelated to nonchelated iron. The resulting increase in oxidative metabolism promotes inflammatory signalling, suggesting that depletion of nonchelated iron in macrophages could lead to an anti-inflammatory phenotype and provide a potential pathway to target in the development of treatment for hypertension [84,87]. Indeed, the administration of therapeutic iron chelators has been shown to both decrease inflammation [88,89] and increase sensitivity to insulin in both animal models of obesity and obese humans [90]. Studies have also shown that the iron chelator deferoxamine reduces pro-inflammatory macrophage signalling in mice, leading to reduced cardiac inflammation and fibrosis [91] and reduced blood pressure in iron-overloaded hypertensive mice [92]. These effects could result from the partial restoration of iron homeostasis in ATMs and adipocytes through an initial reduction in iron overload, followed by increases in mitoNEET expression in ATMs. This would then lead to reduced levels of labile intracellular and mitochondrial iron and thus reduce inflammatory signalling, ameliorating hypertension [7,93–95]. Further research into the link between iron chelation and blood pressure is needed to establish the potential for chelation therapy in alleviating hypertension. This is especially important in the context of obesity since there is a distinct paucity of research linking obesity-induced hypertension to iron overload and chelation.

Overall, these recent findings [7] have shown that the homeostasis of ATM miFe levels is a key factor in regulating the inflammatory phenotype of surrounding adipose tissue, and this balance is significantly impaired in the obese state. As a result, due to the role that PVAT plays in the regulation of arterial tone, it is possible that miFe homeostasis may ultimately act as a regulator of BP through modulation of inflammatory conditions in obesity. The data described in this review also highlight the reciprocal relationship between inflammation and iron sequestration: obesity-induced inflammation leads to iron sequestration as a result of hepcidin upregulation, and excess iron sequestration leads to further pro-inflammatory signalling. The vicious cycle is ultimately incited by obesity and drives hypertension, giving rise to a potential miFe-based treatment approach for obesity-induced hypertension. This treatment could potentially manifest as fat grafts: Joffin *et al.*[7] showed that transplant of ATMs over-expressing mitoNEET (and thus exhibiting depleted miFe) were able to positively alter surrounding adipocyte iron concentration, tissue inflammatory phenotype and systemic metabolism (glucose tolerance and insulin resistance). This discovery forms a promising foundation for the treatment of inflammatory hypertension and should be expanded upon in future studies.

Even in the absence of obesity, several studies have reported an association between serum ferritin levels and risk of hypertension [96–98]. This is likely a result of ROS production, as excess iron has been shown to promote free radical formation [99]. This phenomenon, when present in the vasculature, can lead to inflammation and consequent endothelial dysfunction (ED) and increased BP [100]. This reinforces evidence for the critical role of iron in BP control.

In summary, iron overload has been proven to induce inflammatory signalling in distinct ways in both vasculature and adipose tissue, both of which may lead to hypertension. This justifies the need for further research on the multifaceted influence of iron on inflammation in vascular and adipose tissue, to potentially develop an iron-targeted treatment for inflammatory hypertension.

## SYSTEMIC MAGNESIUM DEPLETION DUE TO REDUCED DIETARY INTAKE RESULTS IN INCREASED BLOOD PRESSURE, INFLAMMATORY CYTOKINE RELEASE AND NOD-LIKE RECEPTOR FAMILY PYRIN DOMAIN-CONTAINING 3 INFLAMMASOME EXPRESSION

Hypertension risk is closely associated with dietary salt intake [101], and multiple studies have reported that this association is a result of inflammation [102–104]. Specifically, high dietary salt intake promotes the formation of the Nod-like receptor family pyrin domain-containing 3 (NLRP3) inflammasome in antigen presenting cells (APCs), which ultimately results in the release of ILs -1β and -18, and in inflammatory hypertension [8,105,106].

**FIGURE 4:**
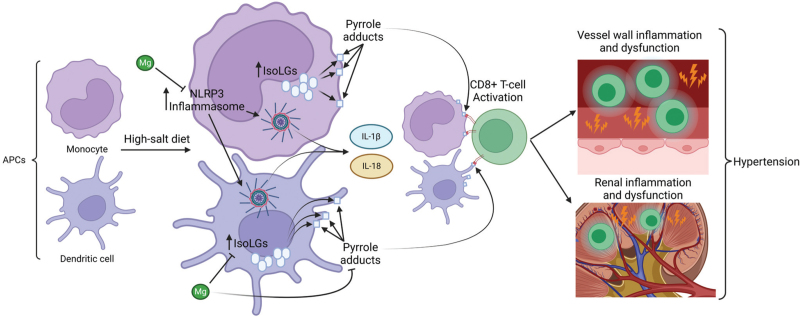
The activation of CD8^+^ T-cells as a result of a high-salt diet and the subsequent hypertension. Monocytes and dendritic cells are two types of antigen-presenting cell (APC). In the presence of high dietary salt, the Nod-like receptor family pyrin domain-containing 3 (NLRP3) inflammasome is upregulated in these cells, and the production of isolevuglandins (isoLGs) as a result of fatty acid peroxidation by reactive oxygen species (ROS) is increased. NLRP3 upregulation leads to increased pro-inflammatory cytokine release (interleukins (ILs) -1β and -18), whilst isoLGs generate pyrrole adducts in the APCs. These adducts are likely presented as neoantigens to CD8^+^ T-cells, activating them. Once activated, T-cells promote inflammation and dysfunction in the vasculature and kidney, leading to increased blood pressure (through mechanisms detailed in Fig. 1). Based on evidence presented by Pitzer Mutchler *et al.*[8], magnesium (Mg) likely inhibits NLRP3 inflammasome activity and isoLG formation, leading to reduced inflammation and subsequent increased blood pressure, although its mechanism of action is not currently confirmed. Figure created with BioRender.com.

Monocytes and dendritic cells are two types of APC that are implicated in inflammation-induced hypertension. As well as releasing pro-inflammatory cytokines that contribute to hypertension, these cells are also involved in elevating BP through the production of isolevuglandins (isoLGs), which are formed through fatty acid peroxidation by ROS [8,107,108]. These isoLGs generate pyrrole adducts in the APCs, which can activate CD8^+^ T-cells, which is likely due to them being presented as neoantigens by the APCs. This process is illustrated in Fig. 4. CD8^+^ T-cell activation in the vasculature and kidney may then give rise to hypertension, since CD8^+^ T-cell activity is integral in the generation of hypertension in some mouse models [109,110]. Recently, evidence was produced by Pitzer Mutchler *et al.*[8] that revealed a major modulatory role for Mg in this inflammatory process.

High levels of inflammation and oxidative stress have been shown to negatively correlate with serum Mg levels, and frequently occur in animals with clinical Mg deficiency [111,112]. Exogenous Mg administration has been shown to reduce the presence of inflammatory markers, decrease the risk of cardiovascular disease (CVD) and lower BP in human patients [112–115]. Mg supplementation has also been recommended in some instances as a dietary adjunctive for the prevention of CVD [116] and is associated with reduced severity of metabolic syndrome (MetS) and thus reduced hypertension [117].

Pitzer Mutchler *et al.*[8] compared BP and inflammatory markers between mice fed a low Mg diet (0.01%), mice fed a control Mg diet (0.08%), and mice fed a high salt diet. Over 5 weeks, the low Mg group exhibited consistently significantly increased systolic BP in comparison to the control group. In both groups, systolic BP was significantly higher after 5 weeks compared to the beginning of the experiment, but the degree of significance was much higher in the low Mg group [8]. Furthermore, the low Mg diet experienced hypomagnesaemia, whilst the control group did not. These findings paper reinforce other papers’ findings regarding the influence of Mg on BP [8,118,119].

Reduced Mg intake resulted in significantly increased serum levels of IL-1β compared to control Mg intake; the inflammatory markers TNF-α and ILs -2 and -10 were also higher but not by a significant margin. This indicates that Mg plays a role in systemic inflammation and thus has the potential to influence arterial hypertension through modulation of inflammatory signalling [8].

In the kidneys, a low Mg diet resulted in significantly increased monocyte infiltration, to a similar level to that seen in the high salt diet. These infiltrating monocytes, however, did not exhibit increased IL-1B, NLRP3 or isoLGs. However, renal dendritic cells expressed significantly higher levels of all three of these inflammatory factors in a high Mg diet compared to a control Mg diet, an effect that was observed in mice fed a high salt diet [8].

Similarly, in the aorta, monocyte levels were increased by a low Mg diet, and these monocytes expressed more NLRP3 and isoLGs than monocytes from mice fed a control diet [8]. In aortic dendritic cells, a low Mg diet increased NLRP3 expression to a similar level as in a high salt diet. Dendritic cells also produced more isoLGs in mice fed a low Mg diet compared to those fed a control Mg diet. Dendritic cells from the aorta of mice with an increased salt intake did not exhibit this increase in isoLGs [8]. These findings suggest that Mg intake has a very similar effect to sodium on blood pressure and inflammatory signalling in the kidney and vasculature, although further research is needed to form a direct comparison between the two micronutrients.

The stimulatory effect of low dietary Mg on dendritic cells was confirmed to be direct, rather than a systemic effect stemming from hypomagnesaemia. When dendritic cells were cultured in a low-Mg environment, the expression of ILs -1β and 18 was increased compared to cells cultured with a control level of Mg [8]. The effect of Mg on the expression of these ILs is dependent upon NLRP3 and caspase-1, demonstrated through the inhibition of NLRP3 and caspase-1, which prevented the increase of ILs -1β and 18 [8].

In the study by Pitzer Mutchler *et al.*[8], the effect of Mg depletion on monocytes and dendritic cells is generally comparable to the effects that result from increased dietary salt intake compared to controls. The link between salt-sensitive hypertension and inflammation is robust [120], and due to the reported similarity of effects between low Mg and high sodium diets, it is highly likely that Mg also plays a role in inflammation-mediated hypertension that is as significant as that of sodium. This potential role is shown in Fig. 4. The observations reported in the study accord well with a study in which decreased serum Mg was associated with increased risk of hypertension [121], and also supports evidence presented in two meta-analyses in which Mg intake was demonstrated to correlate with reduced hypertension risk [117], and in which supplementary Mg led to a dose-dependent reduction in BP in humans, with a more significant effect in those with hypertension [122,123]. Higher systemic Mg levels have also been shown to increase arterial elasticity [124,125]. Due to the close link between inflammation and hypertension, along with evidence covered here reported by Pitzer Mutchler *et al.*[8], it is likely that Mg exerts a beneficial effect on BP through modulation of inflammation via NLRP3, and oxidative stress via isoLG adduct formation, in vascular and renal APCs [8].

## MAGNESIUM SUPPLEMENTATION MITIGATES INFLAMMATORY SIGNALLING AND SYMPTOMS OF THE METABOLIC SYNDROME, INCLUDING HYPERTENSION, IN AN ANIMAL MODEL FOR CHRONIC KIDNEY DISEASE

MetS and CKD are significant risk factors for cardiovascular disease [126]. The conditions, which are both linked to significant vascular alterations respectively affect approximately 10.5% and 12.5% of adults worldwide [127,128]. These alterations are driven by inflammation and the resulting deterioration of endothelial function, resulting in hypertension [129,130]. Hypertension is a major component of MetS, but it is both a cause and an effect of CKD [131]. Consequently, hypertension is a major factor in determining possible morbidities and overall health in patients with MetS and/or CKD. A major role for Mg in inflammatory hypertension in CKD and MetS has recently been reported by López-Baltanás *et al.*[9] and this has been explored in detail below.

**FIGURE 5:**
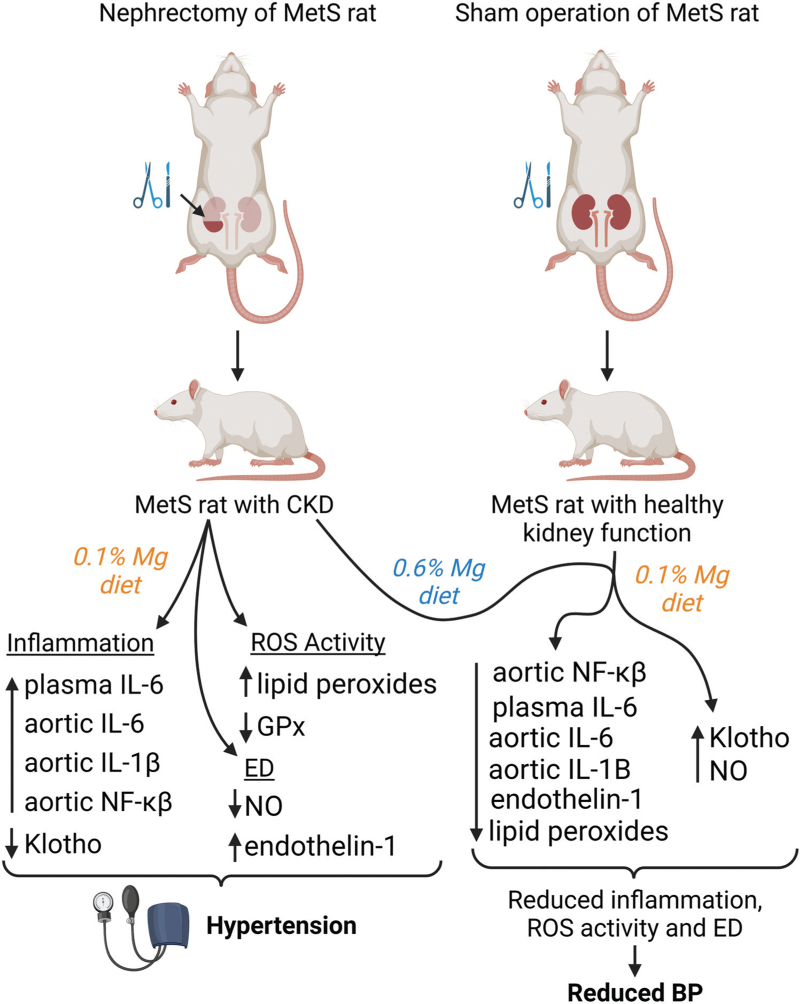
The effects of magnesium (Mg) supplementation on 5/6-nephrectomised rats. Rats underwent surgical removal of a full kidney and 2/3 of the other to generate a model for chronic kidney disease, in which renal failure and symptoms of metabolic syndrome are present. 5/6-nephrectomised rats fed a diet consisting of a standard Mg intake (0.1%) displayed increases in inflammation, ROS activity and endothelial dysfunction (ED) through increased expression of the inflammatory cytokines nuclear factor κβ (NF-κβ), interleukins (ILs) -6 and -1β, as well as lipid peroxides and endothelin-1. Reductions in glutathione peroxidase (GPx), an antioxidant, and nitric oxide (NO) a vasodilator, were also present. Blood pressure itself was increased compared to control sham-operated rats fed a standard Mg diet. On the other hand, 5/6-nephrectomised rats that consumed significantly more Mg displayed levels of these parameters tested by López-Baltanás *et al.*[9] similar to that of sham-operated controls fed a standard Mg diet. GPx expression, was still slightly reduced in Nx-rats, despite being fed a high-Mg diet. Figure created with BioRender.com.

To elucidate the role of Mg in MetS- and CKD-related inflammation, López-Baltanás *et al.*[9] used 5/6-nephrectomised (Nx) rats with a genetic MetS phenotype, a component of which was hypertension, as a model for MetS with CKD. The Nx rats were split into two groups: one fed a diet containing a standard Mg content (0.1%), whilst the other was fed a diet consisting of a high Mg content (0.6%). A visual overview of this procedure is provided in Fig. 5. The positive changes arising from increased Mg intake in Nx rats serve as compelling evidence for the beneficial effects of Mg on inflammatory hypertension.

The major finding from this study is that Nx rats fed a high Mg diet did not exhibit increased blood pressure compared to control rats, whilst Nx rats with lower dietary Mg continued to exhibit elevated BP induced by nephrectomy. Further analysis showed that the maintenance of control-level BP in the high Mg diet group was likely due to reduced inflammatory signalling, as Mg supplementation also maintained levels of NF-κβ, plasma IL-6 and aortic IL-6 and -1β similar to that of controls. These inflammatory markers were all elevated in Nx rats with a standard Mg intake. These data show that despite the presence of renal failure, Mg supplementation reduced markers of systemic and vascular inflammation that contribute to impaired endothelial function and thus to the development of hypertension (Fig. 5) [9]. This highlights the potential of Mg as a treatment for hypertension in MetS and CKD through anti-inflammatory effects.

Standard Mg-diet Nx mice also exhibited reduced Klotho levels, similar to human CKD patients [9,132]. Klotho is a pleiotropic renal protein involved in several physiological processes [133], but importantly, in the context of this review, Klotho modulates chemokine receptor 2-mediated inflammation, which has been shown to contribute to salt-sensitive hypertension in mice [134]. Importantly, a reduction in Klotho means an increase in inflammation. Here, Nx rats fed a high-Mg diet did not exhibit the same reduction in Klotho observed in standard-Mg Nx rats. This suggests that Mg supplementation may ameliorate inflammation via mediation of Klotho expression, and therefore warrants exploring as a promising means for the treatment of hypertension [9].

Nx rats with a standard Mg intake were noted to exhibit reduced levels of glutathione peroxidase (GPx), a family of antioxidants involved in neutralising ROS activity and protecting cells from oxidative damage [135]. However, rats fed an increased Mg diet exhibited only a partial reduction in GPx compared to the standard Mg diet group, indicating that Mg supplementation may protect from oxidative stress. Reduced GPx activity is linked to MetS [9], likely contributing to its hypertension component through reduced protection from oxidative damage and the resulting inflammation [9]. Mg supplementation could therefore ameliorate MetS- and CKD-related hypertension through the restoration of antioxidant activity and prevention of oxidative damage, inflammation, and consequent vascular dysfunction. Lipid peroxides, which also serve as a marker for ROS-induced damage, were significantly raised in Nx rats with a standard Mg intake compared to control rats, whilst rats with high dietary Mg showed no increase in lipid peroxide levels relative to controls. This again reinforces the evidence that increased Mg may decrease oxidative damage, which may then reduce inflammation and improve hypertension.

López-Baltanás *et al.*[9] also revealed a beneficial effect of Mg on levels of the vasoconstrictor endothelin-1 (ET-1) and the vasodilator nitric oxide (NO), both of which are markers of endothelial function and health. Nx rats fed a standard Mg diet exhibited ET-1 levels 170% higher than control, but ET-1 levels in the high-Mg group did not vary from control level. Similarly, NO was significantly decreased in Nx rats with a standard Mg intake, but once again rats with a high Mg intake showed no change in NO compared to controls. This suggests that Mg may reverse some of the changes in endothelial function that contribute to hypertension in MetS.

Separate studies have shown that increased Mg supplementation prevents upregulation of inflammatory cytokines and counteracts increases in ROS production and activation of NF-κβ in an inflammatory environment [136,137]. This evidence strengthens the recent reports from Pitzer Mutchler *et al.*[8] and López-Baltanás *et al.*[9] that suggest Mg may modulate inflammatory conditions in the vasculature with potential subsequent effects on arterial compliance and hypertension.

Mg has been shown to display anti-inflammatory properties by various studies; its role in preventing over-activation of the innate immune response and maintenance of anti-inflammatory conditions has been well documented [138,139,140]. Therefore, the compelling studies by Pitzer Mutchler *et al.*[8] and López-Baltanás *et al.*[9] described here, reinforced by numerous separate reports of Mg-induced reductions in inflammation, can be considered to constitute a solid foundation for a relatively safe and simple method for treating inflammatory hypertension with potentially beneficial off-target anti-inflammatory effects. The two promising updates described here in this paper on the efficacy of Mg-based therapies for inflammatory hypertension indicate that Mg has great potential as an inflammatory modulator, with consequent effects on hypertension. Further research in this niche will be crucial in elucidating the mechanism behind Mg's beneficial effects and developing a novel treatment option for hypertensive patients [8,9].

## SUMMARY

The involvement of the kidneys and vascular and adipose tissue inflammation in hypertension, coupled with the involvement of ROS and a multitude of inflammatory factors, demonstrates the complex and multifaceted nature of inflammation. Due to the complexity of inflammation and its relationship with hypertension, it is unsurprising that various micronutrients should influence the inflammatory process and resulting hypertension. The link between dysregulated iron and Mg homeostasis and inflammation warrants further investigation due to the robust association between inflammation and hypertension. With more advanced research in the field, a new means of ameliorating inflammation and consequent hypertension could emerge. The studies covered in this review solely report findings in animal models, and due to the recency of these results, clinical studies have not yet been able to build on these findings. As a result, the translational importance of the reports detailed in this review is still unknown, but previous clinical data upon which recent studies have built [68,69,141,142] suggest strong potential for a beneficial clinical effect on hypertension.

Therapeutic approaches such as iron chelation therapy, Mg supplementation or a high Mg diet all constitute promising, but still exploratory, possibilities for reducing BP in hypertensive patients. Further research is required to determine the efficacy of these treatment approaches across several clinical settings where hypertension is present. Indeed, clinical studies have shown that increased levels of haemoglobin and transferrin (both iron-bound molecules) are associated with hypertension [143] in humans, so the possibility for the translation of iron-depleting methods suggested by Joffin *et al.*[7] into a clinical setting for the treatment of hypertension is promising. The utility of Mg for the treatment of CVD in humans is also well documented [114–116]. However, due to the complexity of hypertension, which rarely involves inflammation alone but factors such as renal influence and atherosclerosis too, the potential for completely reversing hypertension solely through an inflammation-based treatment is low. As a result, combining an inflammation-targeted treatment, such as Mg supplementation or iron chelation, with one based on enhancing kidney function or targeting the renin-angiotensin-aldosterone system, for example, is much more likely to elicit a significant reduction in BP.

Other micronutrients, such as zinc, folate and B vitamins, have all been shown to influence hypertension, as recent research by Mohtashamian *et al.*[144] and Xiong *et al.*[145] has suggested, though the involvement of inflammation in these studies is unclear. Further research on therapeutic approaches involving iron and Mg, as well as other essential micronutrients, should be carried out in animal models for obesity, CKD and/or MetS to generate more potential avenues for the amelioration of inflammation-related hypertension, as well as in clinical studies to explore the potential benefit of micronutrient-targeted treatments in hypertensive patients.

## ACKNOWLEDGEMENTS

B.J.C. is supported by British Heart Foundation grant AA/18/4/3422, SNS by British Heart Foundation & the Rosetrees Trust project grant PG/22/11044.

Sources of funding: British Heart Foundation AA/18/4/342 and PG/22/11044.

### Conflicts of interest

There are no conflicts of interest.
